# Fine-tuning structural RNA alignments in the twilight zone

**DOI:** 10.1186/1471-2105-11-222

**Published:** 2010-04-30

**Authors:** Andreas Bremges, Stefanie Schirmer, Robert Giegerich

**Affiliations:** 1 Faculty of Technology, Bielefeld University, 33615 Bielefeld, Germany

## Abstract

**Background:**

A widely used method to find conserved secondary structure in RNA is to first construct a multiple sequence alignment, and then fold the alignment, optimizing a score based on thermodynamics and covariance. This method works best around 75% sequence similarity. However, in a "twilight zone" below 55% similarity, the sequence alignment tends to obscure the covariance signal used in the second phase. Therefore, while the overall shape of the consensus structure may still be found, the degree of conservation cannot be estimated reliably.

**Results:**

Based on a combination of available methods, we present a method named *planACstar *for improving structure conservation in structural alignments in the twilight zone. After constructing a consensus structure by alignment folding, *planACstar *abandons the original sequence alignment, refolds the sequences individually, but consistent with the consensus, aligns the structures, irrespective of sequence, by a pure structure alignment method, and derives an improved sequence alignment from the alignment of structures, to be re-submitted to alignment folding, etc.. This circle may be iterated as long as structural conservation improves, but normally, one step suffices.

**Conclusions:**

Employing the tools *ClustalW*, *RNAalifold*, and *RNAforester*, we find that for sequences with 30-55% sequence identity, structural conservation can be improved by 10% on average, with a large variation, measured in terms of *RNAalifold*'s own criterion, the structure conservation index.

## Background

### Introduction

The biological function of an RNA molecule is often determined by the three dimensional structure of the molecule. The structure is often more conserved than the exact sequence of bases in the course of evolution. Therefore, a strong structural consensus among related, but diverged sequences can be taken as an indicator of a preserved functional role.

Following a classification introduced in [1], the structural consensus for a family of RNA molecules can be computed following three different "plans": We can either (A) align the sequences, then fold them jointly, or (B) simultaneously align and fold, or (C) first fold sequences individually, and then align their structures.

**Plan B **is theoretically optimal, as it jointly solves the two optimization problems of alignment and folding under arbitrary scoring functions [2]. However, its computational costs of *O*(*n*^3*m*^) time and *O*(*n*^2*m*^) space, where *n *is sequence length and *m *is the number of sequences, are rather high. Implementations are for example *Foldalign*, *Dynalign*, *PMComp *[3-7], making pragmatic restrictions to improve efficiency.

**Plan C **applies to sequences that are too diverged to be meaningfully aligned based on sequence conservation. First, sequences are folded individually. This must be done with care. One cannot simply compute MFE structures and then align them with a structure alignment program. In an evaluation of 69 sequences from 10 RNA families, the MFE predictions had the same abstract shape (i.e. arrangement of helices) as the consensus structure only in 32 cases (see Table two in [8]). Such lack of consensus in predicted MFE structures makes their structure alignment meaningless - but it does not rule out the existence of a good consensus structure hidden in the near-optimal folding space. Therefore, a representative subset of near-optimal structures must be obtained for each sequence, for example by abstract shape analysis [9]. Then, those structures which have a consensus abstract shape are aligned, for example via tree comparison (*RNAforester *[10,11], *MARNA *[12]). *RNAcast *[8] was the first Plan C approach; a more recent one is *R-Coffee *[13].

**Plan A **is probably the most widely used approach and is the one we are going to strengthen further here. It applies when sequences can be meaningfully aligned using an off-the-shelf multiple sequence alignment tool (e.g. *ClustalW *[14], *T-Coffee *[15], *MAFFT *[16]). Then, the aligned sequences are folded jointly (e.g. *PFOLD *[17], *RNAalifold *[18,19], *ILM *[20], *ConStruct *[21]).

The above listing of Plan A, B, and C methods is far from complete, as the difficulty of the problem is reflected by a large number of approaches. Numerous heuristics have been suggested to retain the power of Plan B approaches, but reduce its high computational cost and overcome its limitation to pairs of sequences, e.g. *MURLET *or *MXSCARNA *[22,23]. The practically minded reader is referred to the *WAR web server for aligning structural RNAs *[24], where presently 14 such methods are on display. Four of them can be categorized as Plan A approaches, which is our concern here. Our contribution presented here should not be considered as yet another approach, but rather, a fine-tuning step which is worthwhile to combine with Plan A methods.

Plan A loses its *raison d'être *when sequence conservation is above 90%. While the sequence alignment is certainly reliable, it carries little extra information compared to folding the sequences individually. The method works best around 75% of sequence similarity. Below 55%, measurements [25] show a decline of performance. This effect has been confrmed in [26] (Figure four). Here, we show how to alleviate this situation to a certain extent. The resulting method is named *planACstar*, as it constitutes an iterated combination of steps from Plan A and Plan C.

### Motivation

**Figure 1:**
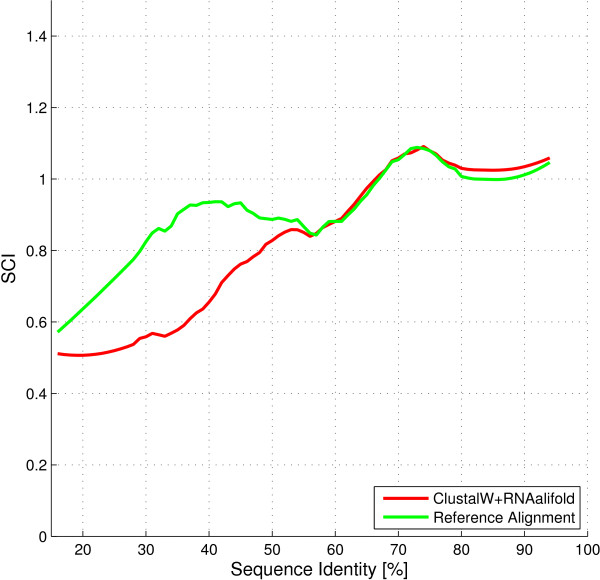
**Performance of Plan A**. Performance of a Plan A approach for RNA families with different levels of sequence identity. The "twilight zone" extends from approx. 55% sequence identity to the left. The reference alignments were taken from BRAliBase. Filtering with a Savitzky-Golay filter was applied afterwards to smoothen the graph.

To evaluate the performance of Plan A in detail, consider Figure 1. The score of Plan A diverges most from the reference alignment in a range of 30-55% sequence identity. In this "twilight zone", the performance of *RNAalifold *drops, which is expressed in a lower structure conservation index (SCI) [27]. The lower index indicates a quality drop of the produced consensus structure, but does not rule out that a better consensus exists, which has not been detected. In fact, this is often the case.

Let us look at the situation in some detail. It is generally known that with increasing divergence, sequence alignment will fail to align bases representing compensatory mutations and carry the covariance signal to be exploited in the subsequent phase. In fact, in the presence of gaps, sequence alignment will *systematically *obscure the covariance signal. As this observation has not been reported in the literature (to the best of our knowledge), let us explicate this phenomenon here.

We use the familiar dot-bracket notation, where paired bases are indicated by matching parentheses, and unpaired bases by dots. For structures ..((....)).. and ..(....).., the expected structure alignment

might show good sequence conservation, two gaps and a compensating base pair change. In any sequence alignment of AAGGAAAACCAA and AACAAAAGA, aligning two bases GG with a single base C, the algorithm has to insert a gap either to the left of C, or to the right. As the resulting score is not affected by this choice, the gap position may be chosen arbitrarily. Let us assume that the alignment algorithm first produces the mismatch, and then inserts a gap to the right of the mismatched base. So far, so good. Exactly the same situation arises in the alignment of CC and G. Again, the algorithm inserts the gap at the right side of G, and aligns the sequences this way:

Now, joint folding of the aligned sequences will produce:

which prevents the second base pair in the upper sequence from being formed: Pairing the second G with the second C would create non-nested base pairing, which is not allowed in the folding algorithm.

### Outline of planACstar

In the 30-55% sequence identity range, we conjecture that the resulting consensus structure is often correct in its overall shape, while structural detail is lost due to the obscured covariance signal. Therefore, we disassemble the alignment and refold the sequences separately, with respect to the preliminary consensus. This separate folding will produce additional base pairs compatible with the consensus. We then align the resulting structures with a *structure *alignment program [10]. This aligns base pairs irrespective of the concrete bases, thus finding compensatory base changes and recovering the covariance signal. Any structure alignment, so obtained, entails a sequence alignment, which we extract. This sequence alignment may now fold into a better consensus than before.

There is a clear limitation in this approach: We may recover base pairs missed in the first alignment folding, but we never undo consensus base pairs which were actually formed in the initial step. These base pairs will always persist in the improved structures, although they may be aligned in a different way. This is why we consider our approach a fine-tuning add-on to Plan A, rather than (yet another) new approach. This is in good analogy to tuning your guitar without reference to a perfect pitch device. You assume that one of the six strings, say A, is on pitch, and tune the other strings to the A string. While your guitar now sounds in harmony, in absolute terms, it may be more out of pitch than before. Our assumption is that, in the twilight zone, the initial alignment will be good enough to point to the string A which is closest to pitch in absolute terms.

This idea has been implemented using the tools *ClustalW*, *RNAalifold*, and *RNAforester*. The quality of the consensus is assessed by *RNAalifold*'s SCI score, the structure conservation index, which according to Gruber et al. [28] is the best measure for structural conservation. Folding an alignment A with *RNAalifold *results in the consensus minimum free energy (MFE) *E*_*A *_as output. *E*_*A *_includes pseudo-energy contributions from observed covariation. Folding the sequences *B*_1_,.. *B*_*n *_separately, we can compute their average MFE  from the separate MFEs. The SCI is the quotient of *EA*/.

Thus, we measure an improvement in terms of *RNAalifold*'s own quality criterion, the achieved SCI.

### Adequacy of SCI improvement

Note that the SCI is not a performance measure in the sense that an alignment optimizing the SCI score is closest to a "true" alignment. It serves as an indicator of structural conservation in diverged sequences, whether or not the conserved structure is "true". Mechanistically, the SCI accounts for the energy required to refold the sequences from their MFE structures into the predicted consensus *X*. It does not preclude the existence of yet another, low energy consensus structure *X'*, which may be structurally quite different and may actually be the relevant one for the RNA's function. Refolding all sequences from their MFE structures into *X' *instead of *X *might require only marginally more energy than with consensus *X*, but again, this does NOT imply that *X *and *X' *are structurally similar. *X' *might be the "true" structure, but Plan A would not notice it at all. Admittedly, the larger the number of sequences considered, and the more diverged they are, the more unlikely is this situation to occur. Plan A approaches could, in principle, be augmented to safeguard against this situation, but only at the high cost of performing suboptimal consensus folding.

Our new approach is based on the premise that it is worthwhile to increase SCI scores. However, we have also evaluated the resulting consensus structures against curated structures, and report on this in the Conclusion and in Additional File 1.

## Results and Discussion

### Algorithm

*planACstar *is an iterated combination of elements of Plan A and Plan C. It uses separate structure predictions, as done in Plan C, but includes information from a multiple sequence alignment and alignment folding as in Plan A. We use *ClustalW *for the initial alignment step, and *RNAalifold *for folding the sequences into the consensus structure, because they are most widely used tools for these tasks in practice. Let *A *be the initial sequence alignment, *C *is the preliminary consensus and *X *is its SCI-score. *C*_*i *_is the projection of the basepairs of the preliminary consensus *C *to a sequence *B*_*i *_from the input set of size *n*, where 1 ≤ *i *≤ *n*. *S*_*i *_is the individual folding of a sequence *B*_*i *_from the input set into the preliminary consensus structure *C*. *Y *is the multiple structure alignment of the folded structures. The multiple structure alignment implies a sequence alignment, which is the improved sequence alignment A* with SCI-score X*.

In pseudocode, the procedure is as follows:

Given a set of RNA sequences *B*_1_,..., *B*_*n*_,

1. *A *← *ClustalW*(*B*_1_,..., *B*_*n*_) - initial sequence alignment

2. *C *← *RNAalifold*(*A*); *X *← sci-score(*C*) - preliminary consensus

3. *C*_*i *_← Projection of basepairs in *C *to *B*_*i *_- (see below)

4. *S*_*i *_← *RNAfold *-C *C*_*i*_(*B*_*i*_) for *i *= 1,.. *n *- individual folding of each *B*_*i *_relative to consensus

5. *Y *← *RNAforester*(*S*_1_,..., *S*_*n*_) - multiple structure alignment

6. A* ← sequence alignment implied by *Y *- extract improved sequence alignment

7. C* ← *RNAalifold*(A*); X* ← sci-score(C*) - fold improved alignment

8. if X* >*X*, set *A *← A* and iterate from step 3, else exit with result *A*, *C *and *X*

The "projection" (Step 3) takes the base pairs from the consensus, removes the gaps with respect to sequence *B*_*i*_, and yields an unsaturated structure *C*_*i *_for *B*_*i*_. The call to *RNAfold *with option -*C C*_*i *_(Step 4) may produce additional base pairs aside from those of *C*_*i*_. These will, in general, be different for each *B*_*i*_.

### Test data

In the evaluation, we used the most recent version of RNAalifold [29] and ClustalW 2.0.11. The SCI was computed with the formula given in [28]. In computing average sequence similarity, we did not use the original BRAliBase computation, but used the improved calculation suggested by Torarinsson et al. [30]. *planACstar *was evaluated on data set 1 of the BRAliBaseII benchmarking database [31]. BRAliBaseII was created in 2005 by Gardner, Wilm and Washietl within a benchmark of multiple sequence alignment programs applied to structural RNAs [25].

Dataset 1 of BRAliBaseII contains various RNA sequences of Group II introns, 5S rRNA, tRNA and U5 spliceosomal RNA. The sequences were obtained from the Rfam database [32]. Each RNA family was chopped into 100 subalignments using a procedure described in [26]. Those subalignments contain 5 sequences each and cover a wide range of sequence identities.

Currently, the full IUPAC code is not supported by *RNAforester*, therefore our method is restricted to concrete RNA sequences as input sequences. This restriction reduces the size of our test data set to 340 out of 388 subalignments.

For comparison, we include the score of the reference alignment. It must be kept in mind that it is scored from the full family alignment. Therefore, the reference alignment does not necessarily represent the optimal subalignment.

### SCI improvements

**Figure 2:**
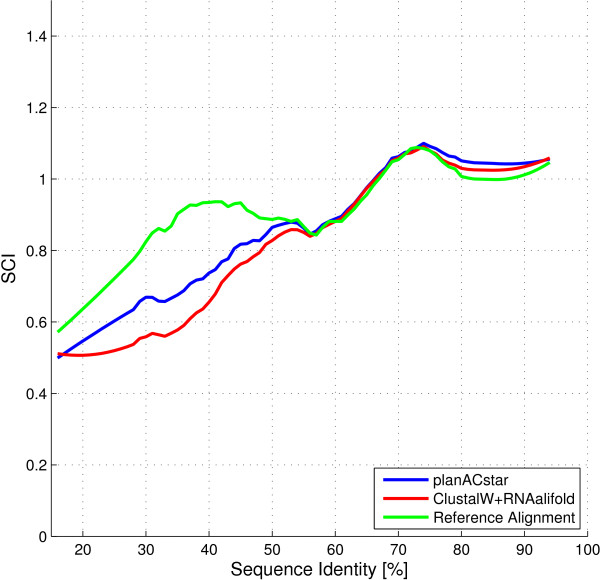
**Performance of planACstar**. Performance of *planACstar *for RNA families with different levels of sequence identity. Refer to Figure 1 for additional information.

In Figure 2, we see the average SCI score at variable sequence similarity. The results of Plan A, *planACstar *and the reference alignment were scored and a filtering with a Savitzky-Golay filter was applied afterwards. The filter uses local polynomial regression to compute a smoothened value for each point.

The results suggest a boundary at 55% sequence similarity. Above 55%, the SCI scores are in agreement, below 55%, the two approaches are outperformed by the reference alignment. This confirms the observation by Gardner, Wilm and Washietl:

*The results suggest that 60% sequence identity is a crude threshold, whereby the structural content of predicted sequence alignments diverges from reference structural alignments*[25].

(The 5% shift of the boundary results from the use of the improved formula for calculating similarity.) Our working hypothesis was that the overall shape of the prediction might still be correct in this twilight zone. In fact, *planACstar *shows an improvement in the zone between 30% and 55% similarity, compared to Plan A. While the underlying RNA sequence is not conserved well, the additional information we extract from its structure improves structure conservation in the overall alignment. Compared to Plan A, *planACstar *reduces the area in the twilight zone to roughly two thirds of its original size.

**Figure 3:**
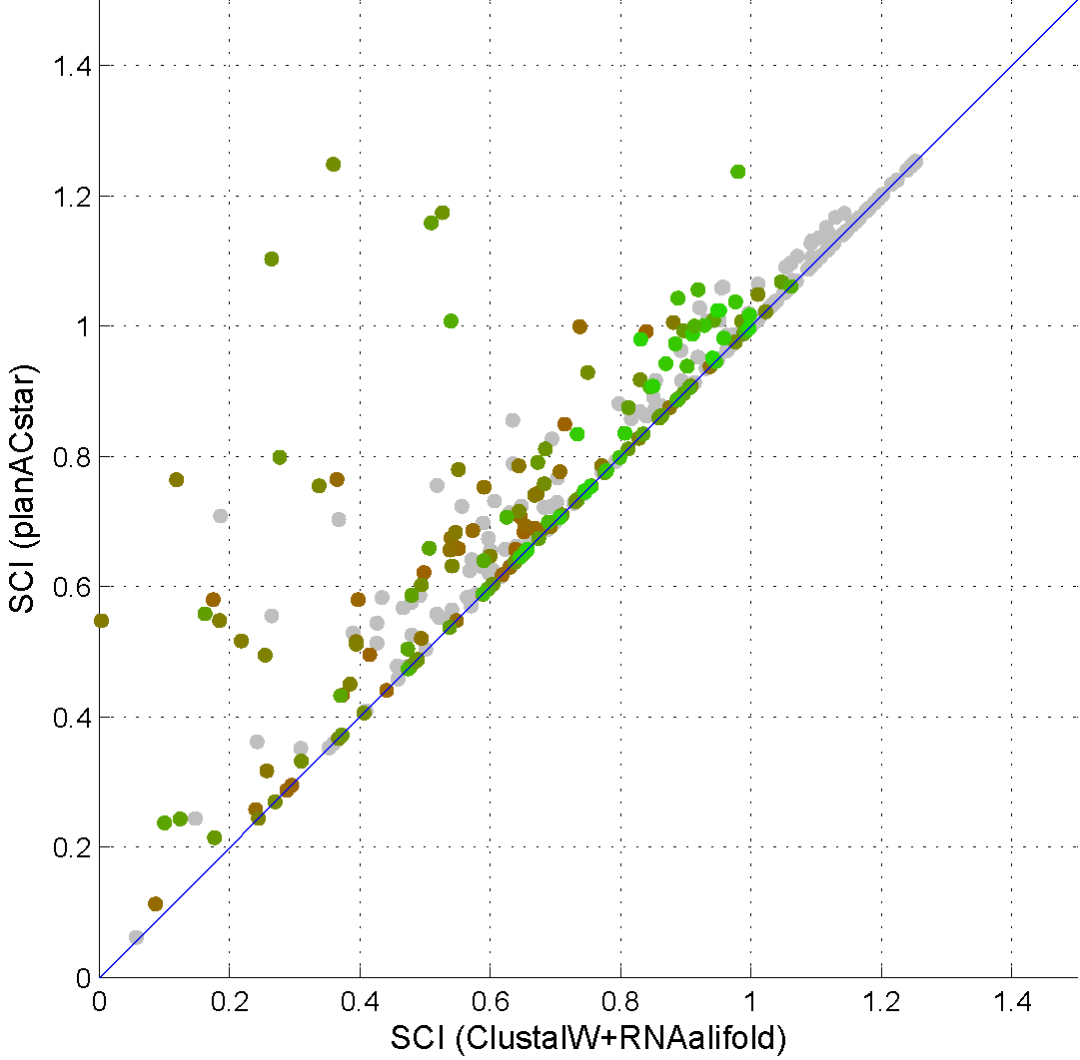
**Dotplot focused on the twilight zone**. SCI-improvement of *planACstar *for RNA families in the "twilight zone", 30-55% sequence identity (color code: green = high similarity, red = low similarity). Grey dots indicate lower or higher sequence identity, i.e. alignments outside of this range.

Figure 3 connects the individual alignments made by *planACstar *and Plan A. Highlighted in color is the interesting sequence identity range, the twilight zone. Note that almost all significant improvements are within this region.

Looking at the full data set, 90% of the dots are on or very close to the diagonal. As expected, above the 55% boundary, our fine-tuning is not necessary. In the twilight zone, we notice that almost 50% of the SCI scores improved.

**Figure 4:**
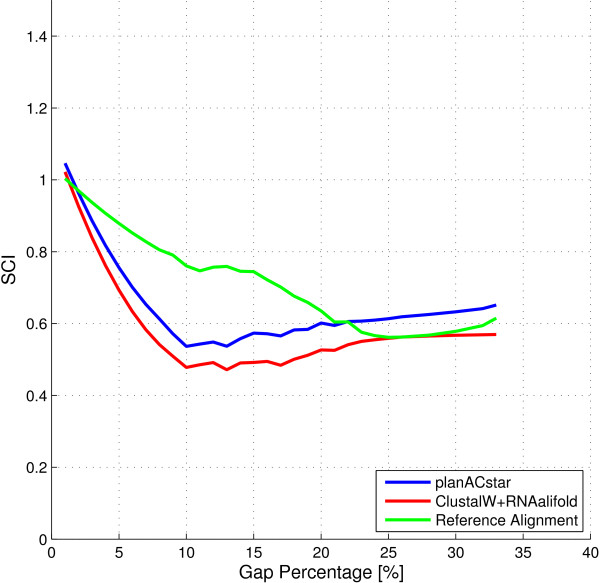
**Correlation between gap content and SCI scores**.

We also tested whether the improvement is related to the gap content in the alignment, but no correlation was observed. This test is documented in Figure 4.

### A detailed observation

Below, a typical path through the pipeline of *planACstar *is shown. As an example, we picked an alignment located in the sequence identity's twilight zone, alignment 83 of the Group II introns. The alignment has a sequence identity of 31% and *planACstar *improves its SCI score from 0.8807 to 1.005.

**Figure 5:**
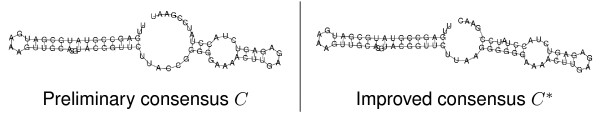
**Improved consensus structure**. Consensus structures *C *and C*. Structure *C *is the preliminary consensus the MFE foldings are restricted to. Structure C* is the improved consensus after the fine-tuning with *planACstar *was applied.

**Figure 6:**
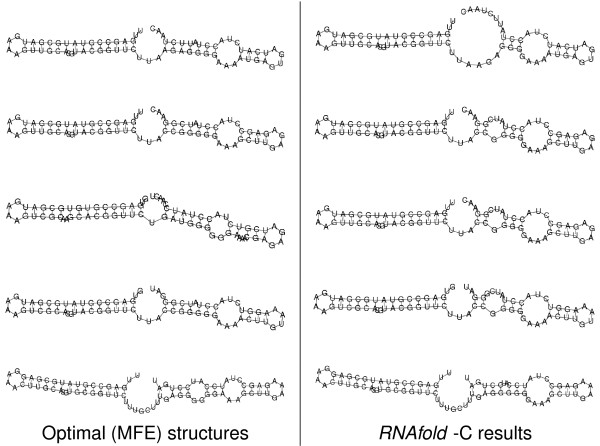
**MFE structures and foldings restricted to the preliminary consensus**. MFE structures versus foldings restricted to the preliminary consensus.

The first step is the initial sequence alignment *A*, conducted by *ClustalW*. The result is the following:

Using *RNAalifold*, its preliminary consensus *C *is calculated. Figure 5 (left) shows the structure of the consensus, scored by *RNAalifold *with -27.92, and a SCI score of 0.8807. The string representation of *C *is the following:

The next step in our pipeline is an individual folding of the single sequences relative to the consensus with *RNAfold *-C. A direct comparison of this procedure to the optimal MFE folding without any consensus is shown in Figure 6. *RNAforester *calculates the multiple structure alignment *Y*, from which an improved sequence alignment A* can be easily extracted. Both are shown below:

Note that there is a new gap at the end of our alignment. The effects on the improved consensus structure C* are shown in Figure 5 (right): the number of basepairs increased. The *RNAalifold MFE*_*A *_improves to -31.88, that is a SCI score of 1.005. A SCI score above 1.0 indicates a very well conserved secondary structure.

### Evaluation summary

Structural conservation in RNA alignments in the twilight zone of 30-55% sequence identity can often be improved. We achieved this by combining already available and widely used RNA tools in the pipeline described above. While the average improvement lies at 10%, a percentage range of improvement cannot be stated, as sometimes, the original SCI value is very close to 0. In the leftmost data point in Figure 3, a structure has two parts, of which the original alignment only allows to identify one. RNAfold finds the second part in each individual sequence, and the structure alignment matches these parts. Hence, the SCI value is raised from (almost) 0 to a significant positive value.

Comparing Figure 1 and 2, we notice that, in the twilight zone, *planACstar *reduced the discrepancy between predictions and reference to about two thirds of its original area. This goes hand in hand with the direct comparison of the SCI scores of *planACstar *and Plan A shown in Figure 3. Almost all improvements occur within the twilight zone.

These improvements are costly to compute. With our data set and particular set of tools employed, we found that *planACstar *is slower than Plan A by a factor of 30 (sequence length 80) to 50 (sequence length 150). To be concrete, the runtime on a single subalignment (*n *= 5, sequence length 150) was increased from 0.297 seconds to 14.896 seconds on a 2 GHz Intel Core 2 Duo processor. This extra efforts results from the individual calls of *RNAfold *for *n *sequences and the multiple alignment of the refolded structures afterwards, where the former effort grows with *O*(*n*), and the latter grows with *O*(*n*^2^). Since each of the pipeline's components may be replaced with a functional equivalent, resource requirements in another implementation may be different. In particular, we are working on a faster version of the pure structure alignment algorithm for the special case when the aligned structures are known in beforehand to share a common overall shape.

On the side, our study also confirmed the recent improvements made to the *RNAalifold *program [29]. Our data were originally computed with the previous version of *RNAalifold*, yielding an average SCI improvement near 20%. With improved gap handling in *RNAalifold*, our fine-tuning makes a smaller improvement of the SCI score. But note that the new *RNAalifold *does not change the alignment, it only scores it more cleverly.

## Conclusions

Our evaluation shows that, with a fairly simple combination of available tools, structure conservation in RNA alignments in the twilight zone can be improved. The degree of achievable improvement varies significantly. In a context of large screening for conserved secondary structures, such as genome-wide RNA gene prediction with *RNAz *[27], Plan A is chosen for its high speed, and using *planACstar *in place of Plan A must be considered too expensive. Moreover, many folded alignments fall outside the twilight zone, and no improvement is to be expected from fine-tuning anyway. However, once certain alignments have been determined as promising and to be are used for model building, we suggest that fine-tuning with *planACstar *should be applied. Our implementation is available on the Bielefeld Bioinformatics Server at http://bibiserv.techfak.uni-bielefeld.de/planACstar/.

While we have built our evaluation on the three tools *ClustalW*, *RNAalifold *and *RNAforester*, note that each of these constituents may be replaced by a functional equivalent to create another instance of *planACstar*. Recovering the covariance signal obscured by sequence alignment remains the underlying general idea.

While often interpreted in this way, a high SCI does not necessarily imply that the associated structure is close to the truth. It is not known currently to what an extent this interpretation is valid, in particular within the twilight zone. We have compiled a second test data set from curated structures, taking their consensus as a standard of truth. We created 80 data sets of 5 sequences each, randomly selected from the Szymanski 5S Ribosomal RNA database [33], taking care that the corresponding subaligments fall within the twilight zone. In 55 (of 80) cases, *planACstar *either confirmed the initial prediction (23 cases), or it improved the SCI and also made the alignment more similar to the reference (22 cases). In 28 cases, the SCI was improved but the alignment became less similar to the reference.. In 26 cases, this was due to moderate local rearrangement of base pairs, while the overall structure was retained. In one case, we found that *planACstar *was fine-tuning to the wrong string, and in the other remaining case, one (correct) helix was strengthened on account of the other (correct) helix, with an overall negative effect. Details of this study are given in Additional File 1, and the complete data set in Additional File 2. There, we also look at the worst case in detail, and show how the pipeline performs when applied to the "true" alignment. As a final observation, we note that there were 7 cases where the alignment improved whereas the SCI did not (and our pipeline hence reports the original SCI and alignment). This indicates that there are a few extra cases where fine-tuning could improve the predicted consensus structure. However - since in practice we have no reference structure available - we have no criterion to take advantage of this fact.

## Authors' contributions

RG and SS designed the study. AB implemented the *planACstar *pipeline and performed the first evaluation. SS performed the evaluation of the SCI improvement against reference structures. Both AB and SS contributed equally to this work and should be considered as joint first author. All three cooperated closely on the manuscript. All authors read and approved the final manuscript.

## Supplementary Material

Additional file 1 **adequacy of SCI improvement**. This supplement reports on an evaluation which uses curated reference structures to evaluate whether an improvement of the SCI is linked to bringing a predicted consensus structure closer to the reference. Click here for file

Additional file 2 **archaea subalignments**. This file provides the subalignments of archaea sequences created and used in the study described in Additional File 1. This is a zipped archive of files in Fasta format. Click here for file
