# Supplementary material for: Fine-tuning structural RNA alignments in the twilight zone
Source: BMC Bioinformatics. 2010 Apr 30;11:222. doi: 10.1186/1471-2105-11-222 (PMC2876130; doi:10.1186/1471-2105-11-222)
Supplement: Additional file 1 — adequacy of SCI improvement. This supplement reports on an evaluation which uses curated reference structures to evaluate whether an improvement of the SCI is linked to bringing a predicted consensus structure closer to the reference. [file 1471-2105-11-222-S1.PDF]

1 Relating SCI-based predictions to a reference structure - 5S Ribosomal RNA

1.1 Setting up the evaluation scenario

As explained in the main text, *planACstar* either improves an alignment of RNA sequences towards a larger SCI value, or else it leaves alignment and SCI unchanged. The question may be raised whether improving the SCI is a worthwhile thing to do. The SCI, by itself, measures a physical property of a set of sequences. It indicates a possible consensus structure in a favorable energy range, where, aside from folding energy, the SCI also contains a pseudo-energy contribution to capture sequence covariance. The SCI is used when reference data is not known, as an indicator that there is structural conservation, and with a certain chance that the predicted consensus structure is a relevant one in nature. Still, a high SCI value does not rule out a quite different functional structure, which is also conserved.

If you are used to routinely trust the SCI, you may be surprised by such skepticism. But think of the following exercise: Start with a single sequence s_1 and execute Plan A on it. You obtain the MFE structure X_1 which is also the consensus as there is only one sequence. $SCI(X_1) = 1$. Now add a second sequence s_2, execute Plan A on both, obtain consensus structure X_2 with $SCI(X_2)$, probably also close to 1. Continuing this for a while, you obtain structures $X_1, X_2, \ldots, X_k$ with the structure still changing in each step. The important thing to notice is that the structure X_k – which we tend to trust more than its predecessors as it is based on more data – has already existed in the suboptimal folding space of all earlier computations, which did not yet contain sequence S_k. The better or even "true" structure X_k has always existed as a good consensus candidate, but has gone unnoticed.

The SCI has been evaluated extensively as an indicator of structural conservation, and has performed superior to a dozen of other computational criteria in [1]. Still, one may wonder whether the particular SCI improvement achieved by *planACstar* actually changes the predicted structure in the right direction.

By such considerations, we were led to an evaluation of the structural change, which comes along with SCI improvement by *planACstar*, against a trusted reference structure. As reference data, we selected high quality RNA alignment data and consensus structure information from the Szymanski 5S Ribosomal RNA Database [2, 3].

How to measure improvement? One could either measure the similarity of the reconstructed sequence alignment to the reference, or the similarity of the predicted consensus structure to the reference structure. Given the consensus structure, the supporting alignment still allows for considerable arbitration in detail, for example with respect to the placement of gaps. We cannot expect that two different methods make these decisions in the same way, and we cannot discern spurious from true differences. Since the alignment is mainly introduced to support the consensus, we decided to compare directly the consensus structures in the predictions and the reference.

To generate test sets, we select from the database 80 subalignments of 5 sequences each, after omitting sequences with general IUPAC codes, as our pipeline currently does

not support full IUPAC codes. The selection is randomized, since in the database, the sequences are ordered according to phylogeny, and choosing contiguous subalignments would not yield test data in the twilight zone. As we construct subalignments, we may get columns in the subalignment which consist of gaps only. These columns arise from extra information in the bigger alignment we created the subalignment from. Thus, we cut out all gap-only columns from each subalignment and also adjust the reference consensus in this way for each subalignment. This yields the reference structure R for each unaligned data set. To obtain unaligned sequences as input to *planACstar*, we then remove all gaps from the sequences in the subalignment, leaving its consensus structure intact.

On these data sets, we run two experiments:

- *Experiment A:* What is the relationship between reference, the SCI score and the consensus predicted by *RNAalifold*? To this end, we want to start our pipeline on the reference *alignment*. We use a modified version of our pipeline which skips the initial sequence alignment step (using the reference alignments instead), and also reports SCI-changes to the worse. Questions are: How close is the consensus assigned by RNAalifold, when given the correct alignment, to the reference consensus? Is the SCI optimal or can it be improved via our pipeline? If so, does the predicted consensus move back towards the reference structure, or farther away from it?

- *Experiment B:* Here we use our unmodified pipeline, applying it to unaligned sequences as it happens in practice. Questions are: How well does the method overall approximate the consensus alignment, and in particular, does our SCI improvement also imply a step towards a predicted consensus that is more similar to the reference.

In both experiments, we run the modified/unmodified *planACstar* on all data sets. Each run returns an initial predicted consensus structure X_1, and an (hopefully) "improved" consensus X_2, with $SCI(X_2) \geq SCI(X_1)$. Remember that *planACstar* leaves things unchanged if the SCI cannot be improved. But here, for the sake of evaluation, we record the changed structure X_2 in either case. In order to find out whether our method brings us closer to the reference R, the task is to compare X_1 and X_2 to R. Note that they are consensi with no specific sequence information associated. We compare them purely as structures. Base pair metrics are inadequate, as the structures can be of different size. We compute their relative similarity score σ_{rel} by pure structure alignment as implemented in *RNAforester*. For structures x, y we have $L < \sigma_{rel}(x, y) \leq 1$, where $\sigma_{rel}(x, y) = 1$ if and only if $x = y$. Dissimilar structures get negative scores, where the lower bound L depends on structure size and parameter settings, with $L \approx -3.44$ for our data sets. (For scoring details see [4]). Finally, $\sigma_{rel}(X_2, R) > \sigma_{rel}(X_1, R)$ is taken as evidence that the improvement of the SCI has actually improved the consensus structure.

1.2 Caveats

Two caveats must be kept in mind with a study like this. Caveat 1 regards to the fact that we are constructing subalignments of a very large alignment, too large to apply our pipeline to it. (We may run *RNAalifold* on the complete alignment – which returns a global SCI score of 1.266877 – but structure alignment is (currently) not feasible for 80 or more structures.) The subalignments still contain certain arrangements of bases that are made in their special way *because* of other members of the large alignment, not present in the subalignment. There is no way to reconstruct this information just given the sequences in the subalignment. In the following tables, we therefore include the number of gap-only columns that had to be removed, as they are reminiscents of this situation.

Caveat 2 is of a deeper nature: A consensus structure has no existence in nature – it is a human construct designed to elucidate for us the essential structural features of a certain functional class of RNA. *In vivo*, each RNA molecule operates as an individual and may have additional structural features compared to the consensus.

1.3 Experiment A: Evaluation on Archaea subalignments

Simply said, in this experiment we try to improve our chosen "truth". We can only make things worse – but how often and how much? From our 5 groups of 16 data sets each, we choose Group 3 in Figure 1 for discussion, because it contains the worst case that is also discussed in detail later.

First, we observe from column $\sigma_{rel}(X_1, R)$ that *RNAalifold* never predicts the exact reference structure from the reference alignment! This is true also for the other data sets. On the other hand, the assigned consensus is quite good with σ_{rel} values close to 1. Our second observation is quite reassuring – *planACstar* does not tend to change the reference alignment, except for data set 0011-0015, which we study in detail later.

1.4 Experiment B: Evaluation on Archaea sequences

This experiment uses the unmodified *planACstar* pipeline, running the dis-aligned sequences through ClustalW as the first step. Results are shown in Figure 2.

Since the sequence sets are the same as in the previous table, let us first compare the SCI and similarity scores. As to be expected, SCI scores are lower for the ClustalW alignments and predicted structures are more distant from the reference. Now let us turn to the performance of *planACstar* in this scenario: The outcome can be partitioned into four situations:

- *improved* means that an SCI improvement was found and the predicted consensus was also improved, has stayed the same, or has changed but retained its degree of similarity with the reference. These cases are labeled (better,better) or (same,better) in the rightmost columns.

Figure 1: Experiment A: *planACstar* applied to reference alignments (data set 3)

Input sequences	Mean pairwise ID, Torarinsson	SCI score after first step	$\sigma_{rel}(X_1, R)$	$\sigma_{rel}(X_2, R)$	removed gap-only columns	length of R	length of X_1	length of X_2	Similarity score improvement	SCI improvement
0001-0005	0.585433	0.870124	0.852804	0.586957	13	136	136	132	worse	worse
0006-0010	0.685626	0.914230	0.805288	0.805288	17	132	132	132	same	same
0011-0015	0.698805	0.835383	0.0206897	-0.17695	14	135	135	132	worse	better
0016-0020	0.561589	0.855754	0.653302	0.489441	13	136	136	133	worse	worse
0021-0025	0.605751	1.001347	0.851765	0.529691	16	133	133	133	worse	worse
0026-0030	0.637686	1.069746	0.875	0.759582	13	136	136	133	worse	worse
0031-0035	0.597336	1.043172	0.896074	0.637363	16	133	133	134	worse	worse
0036-0040	0.621625	1.092444	0.801762	0.64588	15	134	134	132	worse	worse
0041-0045	0.604924	1.053442	0.851765	0.662592	16	133	133	133	worse	worse
0046-0050	0.634963	0.866269	0.734266	0.592506	16	133	133	129	worse	worse
0051-0055	0.635476	1.077000	0.797297	0.655052	17	132	132	129	worse	worse
0056-0060	0.577462	1.072558	0.814978	0.635641	11	138	138	135	worse	worse
0061-0065	0.588971	0.806718	0.873832	0.653416	17	132	132	129	worse	worse
0066-0070	0.583040	1.062100	0.937063	0.650814	12	137	137	134	worse	worse
0071-0075	0.604021	1.048182	0.892601	0.605917	14	135	135	134	worse	worse
0076-0080	0.603763	1.055271	0.874126	0.630037	16	133	133	134	worse	worse

- *no change* is reported by *planACstar* – and rightfully so – when neither SCI nor structure improves. This is indicated by the labels (worse,worse), (worse,same), (same,same), and (same,worse).
- *blind alley* occurred when the SCI was improved, but the structure actually changed to the worse. This happens when the initial ClustalW alignment suggests a consensus which is essentially wrong, and *planAcstar* fine-tunes to the wrong suggestion. This situation is labeled (worse,better).
- *chance missed* can be stated when *planACstar* reports no change, but the re-alignment with *RNAforester* has actually made the consensus more similar to the reference. This is case, labeled (better,same) or (better,worse), must go unnoticed in practice, when we do not have a reference structure to compare to.

7 out of the 9 possible situations occur within data set 3, and 8 of them overall.

In Figure 2, most cases fall in the first two categories, *improved* and *no change*, where *planAstar* behaves as it should. We see three *blind alleys* and 4 *chance missed*, among them data set (0011-115).

Summing up over all data sets, we observe the outcome shown in the following table:

Figure 2: Experiment B: *planACstar* applied to ClustalW alignments (data set 3)

Input sequences	Mean pairwise ID, Torarinsson	SCI score after first step	$\sigma_{rel}(X_1, R)$	$\sigma_{rel}(X_2, R)$	removed gap-only columns	length of R	length of X_1	length of X_2	Similarity score improvement	SCI improvement
0001-0005	0.585433	0.805692	0.662679	0.620209	13	136	132	133	worse	worse
0006-0010	0.685626	0.882169	0.805288	0.732673	17	132	132	132	worse	worse
0011-0015	0.698805	0.546985	-0.489157	-0.407018	14	135	135	136	better	same
0016-0020	0.561589	0.581219	0.603248	0.610209	13	136	134	134	better	better
0021-0025	0.605751	0.654180	0.305882	0.368978	16	133	128	142	better	better
0026-0030	0.637686	0.815246	0.801394	0.728538	13	136	133	134	worse	better
0031-0035	0.597336	0.701394	0.792727	0.623116	16	133	132	135	worse	worse
0036-0040	0.621625	0.949143	0.714129	0.715232	15	134	131	132	better	worse
0041-0045	0.604924	0.800367	0.737095	0.717949	16	133	132	134	worse	better
0046-0050	0.634963	0.583073	0.518171	0.272838	16	133	128	128	worse	better
0051-0055	0.635476	0.872414	0.589831	0.706271	17	132	129	129	better	better
0056-0060	0.577462	0.407408	0.391252	0.472255	11	138	133	133	better	better
0061-0065	0.588971	0.713228	0.66747	0.686131	17	132	130	130	better	worse
0066-0070	0.583040	0.718022	0.768675	0.681764	12	137	133	134	worse	worse
0071-0075	0.604021	0.854003	0.696158	0.72093	14	135	132	133	better	worse
0076-0080	0.603763	0.518480	0.667526	0.25	16	133	131	147	worse	worse

Situation	σ_{rel}, SCI	count
improved		22
	same, better	3
	better, better	19
no change		23
	same, worse	2
	same, same	1
	worse, same	0
	worse, worse	20
blind alley	worse, better	28
chance missed		7
	better, worse	5
	better, same	2

Complete evaluation data are shown in Section 2 of this supplement. The complete set of $5 * 16$ data sets is available as the supplementary file archaea_subalignments.zip.

1.5 Detailed view on data set 3, (0011-0015)

The most “difficult” of our randomized data sets appears to be data set (0011-0015) in data set 3.

Data set 3 (0011-0015) in Experiment A Fed with the reference alignment, *RNAalifold* folds two tight hairpins in the left part of the alignment, where the reference has one hairpin with large loops and bulges. In other words, reference and prediction have different abstract shapes. The two small hairpins happen to appear in all five sequences, and hence are predicted conserved. The structure alignment of the predicted consensus to the reference requires numerous gaps in both structures. In particular, the left small hairpin is considered an insertion, while the right one is aligned to the inner part of the long single hairpin in the reference. With the rest of the structure aligning quite well, the score is close to zero.

```
Structure alignment by RNAforester
global optimal score: 9
relative score        0.0206897

 reference consensus      .((((.((((((((....(((((((...------------.-...((((((....
 RNAalifold consensus     ...((.((((((((....((----(((((((......))).))))..((((----
                          *  *****************    *               *      ****
 reference consensus      .........))))..))....)))))).))....(((((.....((((((.((..
 RNAalifold consensus     ---(....))))).--............))....(((((.....((((((.((..
                              **** *****   *****     ****************************
 reference consensus      ..))))))))....)))))...)))))).))))))...
 RNAalifold consensus     ..))))))))....)))))...)))))).)))).....
                          *********************************  ***
```

Applying one iteration of *planACstar*, the alignment is somewhat compacted, the SCI improves, but the structure moves farther away from the reference, with the similarity score turning negative (-0.1769, Figure 1). Recalling the explanation in the main text: Since the individual folding of the sequences is constrained by the consensus predicted from the first round, the incorrect shape of the ClustalW-based prediction is the *blind alley* we cannot escape from. So much for *RNAalifold's* view of the reference alignment.

Data set 3 (0011-0015) in Experiment B On a ClustalW alignment of this data set, we can expect things to get worse. The initial similarity score is negative already (-0.489157, Figure 2).

```
Structure alignment by RNAforester
global optimal score: -203
relative score:         -0.489157

 reference consensus     .((((.((((((((....(((((((.......((((((.............))))
 RNAalifold consensus    .......((((((....---------------(((((((((......))).))))
                         *    * ****** ***               ******   ******   *****
 reference consensus     ..-------------------)).....))))).))....(((((.....(((((
 RNAalifold consensus    ..(((((....))))).....))..................((((.....(((((
```

```
                        **                   *******     *  **** **************
reference consensus     (.((....-))))))))....)))))...)))))).))))))...
RNAalifold consensus    (.((.....))))))))....))))-....)))))-)---.....
                        ******** **************** *** ***** *     ***
```

Fed with dis-aligned sequences, re-aligned by ClustalW, the alignment allows for fewer basepairings overall. We obtain the same difference in terms of abstract shape, and find ourselves in another *blind alley*. When applying *planACstar* to the initial prediction, the alignment improves a bit (score -0.407018, Figure 2), mainly near the 3' end (data not shown). But the SCI comes out the same, and amusingly, this case constitutes a *chance missed* inside a *blind alley*.

2 Complete evaluation data

2.1 Dataset 1

Figure 3: Measurements on dataset 1

Input sequences	Mean pairwise ID, Torarinsson	SCI score after first step	$\sigma_{rel}(X_1, R)$	$\sigma_{rel}(X_2, R)$	removed gap-only columns	length of R	length of X_1	length of X_2	Similarity score improvement	SCI improvement
0001-0005	0.575394	0.363383	0.401425	0.453933	11	138	136	136	better	better
0006-0010	0.638084	0.779584	0.688172	0.621429	13	136	133	136	worse	worse
0011-0015	0.517007	0.257139	0.106383	0.0660737	14	135	137	148	worse	better
0016-0020	0.662737	0.872217	0.538462	0.577972	13	136	132	133	better	better
0021-0025	0.780263	0.919589	0.759124	0.759124	22	127	127	127	same	same
0026-0030	0.622604	0.723929	0.621287	0.647482	15	134	130	132	better	better
0031-0035	0.659326	0.891304	0.480181	0.505051	13	136	131	131	better	better
0036-0040	0.577202	0.609400	0.631206	0.604811	12	137	133	136	worse	better
0041-0045	0.661753	0.953634	0.706714	0.647465	20	129	128	131	worse	better
0046-0050	0.553618	0.617821	0.731884	0.587952	14	135	133	135	worse	worse
0051-0055	0.614517	0.729748	0.70037	0.463476	15	134	133	132	worse	worse
0056-0060	0.619591	0.576790	0.472527	0.658683	20	129	127	130	better	better
0061-0065	0.682189	0.870900	0.804819	0.804819	18	131	131	131	same	better
0066-0070	0.667464	0.882401	0.672269	0.636804	16	133	132	133	worse	better
0071-0075	0.672379	0.821675	0.725275	0.723971	14	135	132	131	worse	better
0076-0080	0.537840	0.677805	0.57243	0.565321	11	138	134	136	worse	worse

2.2 Measurements on dataset 2

Figure 4: Dataset 2

Input sequences	Mean pairwise ID, Torarinsson	SCI score after first step	$\sigma_{rel}(X_1, R)$	$\sigma_{rel}(X_2, R)$	removed gap-only columns	length of R	length of X_1	length of X_2	Similarity score improvement	SCI improvement
0001-0005	0.633686	0.593113	0.673418	0.673418	17	132	130	130	same	worse
0006-0010	0.660354	0.654670	0.604396	0.588364	17	132	130	131	worse	worse
0011-0015	0.647854	0.828547	0.65942	0.606557	17	132	128	130	worse	worse
0016-0020	0.665799	0.779362	0.776978	0.690391	19	130	128	129	worse	worse
0021-0025	0.616680	0.644982	0.773653	0.723353	15	134	133	133	worse	better
0026-0030	0.662720	0.871288	0.819026	0.734091	22	127	127	129	worse	better
0031-0035	0.680935	0.898117	0.668966	0.644419	13	136	134	133	worse	better
0036-0040	0.603338	0.789633	0.546075	0.229167	12	137	134	135	worse	worse
0041-0045	0.645979	0.595046	0.535109	0.654676	15	134	132	132	better	better
0046-0050	0.587465	0.695834	0.530612	0.514223	14	135	131	131	worse	better
0051-0055	0.591039	0.610852	0.548043	0.525581	9	140	135	136	worse	better
0056-0060	0.621948	0.565641	0.352941	0.294554	17	132	129	140	worse	better
0061-0065	0.537464	0.722275	0.652124	0.593458	12	137	134	135	worse	better
0066-0070	0.744668	0.876705	0.843672	0.843672	22	127	127	127	same	better
0071-0075	0.627956	0.661746	0.792593	0.76808	19	130	128	128	worse	better
0076-0080	0.619971	0.905755	0.668172	0.645235	12	137	133	134	worse	better

2.3 Dataset 3

Figure 5: Measurements on dataset 3

Input sequences	Mean pairwise ID, Torarinsson	SCI score after first step	$\sigma_{rel}(X_1, R)$	$\sigma_{rel}(X_2, R)$	removed gap-only columns	length of R	length of X_1	length of X_2	Similarity score improvement	SCI improvement
0001-0005	0.585433	0.805692	0.662679	0.620209	13	136	132	133	worse	worse
0006-0010	0.685626	0.882169	0.805288	0.732673	17	132	132	132	worse	worse
0011-0015	0.698805	0.546985	-0.489157	-0.407018	14	135	135	136	better	same
0016-0020	0.561589	0.581219	0.603248	0.610209	13	136	134	134	better	better
0021-0025	0.605751	0.654180	0.305882	0.368978	16	133	128	142	better	better
0026-0030	0.637686	0.815246	0.801394	0.728538	13	136	133	134	worse	better
0031-0035	0.597336	0.701394	0.792727	0.623116	16	133	132	135	worse	worse
0036-0040	0.621625	0.949143	0.714129	0.715232	15	134	131	132	better	worse
0041-0045	0.604924	0.800367	0.737095	0.717949	16	133	132	134	worse	better
0046-0050	0.634963	0.583073	0.518171	0.272838	16	133	128	128	worse	better
0051-0055	0.635476	0.872414	0.589831	0.706271	17	132	129	129	better	better
0056-0060	0.577462	0.407408	0.391252	0.472255	11	138	133	133	better	better
0061-0065	0.588971	0.713228	0.66747	0.686131	17	132	130	130	better	worse
0066-0070	0.583040	0.718022	0.768675	0.681764	12	137	133	134	worse	worse
0071-0075	0.604021	0.854003	0.696158	0.72093	14	135	132	133	better	worse
0076-0080	0.603763	0.518480	0.667526	0.25	16	133	131	147	worse	worse

2.4 Dataset 4

Figure 6: Measurements on dataset 4

Input sequences	Mean pairwise ID, Torarinsson	SCI score after first step	$\sigma_{rel}(X_1, R)$	$\sigma_{rel}(X_2, R)$	removed gap-only columns	length of R	length of X_1	length of X_2	Similarity score improvement	SCI improvement
0001-0005	0.618600	0.661451	0.587678	0.709677	14	135	133	133	better	better
0006-0010	0.644533	0.776447	0.808057	0.721578	19	130	130	132	worse	better
0011-0015	0.724371	0.805471	0.678436	0.614815	20	129	128	129	worse	better
0016-0020	0.732050	0.928399	0.737864	0.737864	17	132	132	132	same	worse
0021-0025	0.611627	0.578307	0.798561	0.674208	15	134	132	134	worse	better
0026-0030	0.641667	0.850855	0.677686	0.626667	16	133	130	135	worse	better
0031-0035	0.629927	0.561591	0.425373	0.535211	14	135	133	133	better	better
0036-0040	0.587735	0.665083	0.467416	0.482286	9	140	134	135	better	worse
0041-0045	0.564555	0.574642	0.451467	0.541254	12	137	133	132	better	better
0046-0050	0.546582	0.590826	0.516373	0.41779	14	135	131	135	worse	worse
0051-0055	0.576492	0.595165	0.453271	0.384793	10	139	141	145	worse	better
0056-0060	0.679148	0.814056	0.784933	0.741191	17	132	131	131	worse	worse
0061-0065	0.694296	0.825381	0.634146	0.591241	18	131	129	131	worse	worse
0066-0070	0.576205	0.435633	0.46022	0.487805	19	130	127	130	better	better
0071-0075	0.620459	0.879074	0.823529	0.776744	15	134	133	134	worse	better
0076-0080	0.593731	0.763676	0.742656	0.743259	15	134	133	135	better	better

2.5 Measurements on dataset 5

Figure 7: Dataset 5

Input sequences	Mean pairwise ID, Torarinsson	SCI score after first step	$\sigma_{rel}(X_1, R)$	$\sigma_{rel}(X_2, R)$	removed gap-only columns	length of R	length of X_1	length of X_2	Similarity score improvement	SCI improvement
0001-0005	0.640867	0.732851	0.736462	0.592857	18	131	132	133	worse	worse
0006-0010	0.590282	0.458442	0.596154	0.654249	11	138	134	137	better	worse
0011-0015	0.571918	0.672297	0.638596	0.498824	12	137	134	137	worse	worse
0016-0020	0.689565	0.750110	0.803398	0.778182	21	128	128	129	worse	better
0021-0025	0.612344	0.456927	0.652709	0.609013	17	132	128	129	worse	worse
0026-0030	0.547838	0.367163	0.430894	0.314793	15	134	132	143	worse	better
0031-0035	0.618242	0.616218	0.628571	0.628571	15	134	130	130	same	better
0036-0040	0.615034	0.593427	0.568371	0.64311	15	134	131	131	better	better
0041-0045	0.613712	0.606577	0.494545	0.4	14	135	130	132	worse	worse
0046-0050	0.654900	0.845885	0.745995	0.834437	16	133	133	133	better	better
0051-0055	0.674954	0.895678	0.739884	0.807649	16	133	132	132	better	better
0056-0060	0.589221	0.725692	0.603509	0.530035	13	136	135	137	worse	better
0061-0065	0.607257	0.746502	0.703529	0.755102	15	134	132	132	better	better
0066-0070	0.630004	0.911804	0.752427	0.665083	20	129	127	129	worse	better
0071-0075	0.669437	0.838100	0.769793	0.79562	21	128	125	126	better	same
0076-0080	0.607016	0.759102	0.511312	0.48	9	140	136	136	worse	better

References

[1] Gruber AR, Bernhart SH, Hofacker IL, Washietl S: **Strategies for measuring evolutionary conservation of RNA secondary structures**. *BMC Bioinformatics* 2008, **9**:122+.

[2] Szymanski M, Barciszewska MZ, Erdmann VA, Barciszewski J: **5S Ribosomal RNA Database.** *Nucleic Acids Res* 2002, **30**:176–178, [[http://view.ncbi.nlm.nih.gov/pubmed/11752286]].

[3] Szymanski M, Barciszewska MZ, Erdmann VA, Barciszewski J: **5S Ribosomal RNA Database**. *[http://www.man.poznan.pl/5SData/].*

[4] Höchsmann M, Voss B, Giegerich R: **Pure Multiple RNA Secondary Structure Alignments: A Progressive Profile Approach**. *IEEE/ACM Transactions on Computational Biology and Bioinformatics* 2004, **1(1)**:53–62.
